# Immunity to Cytomegalovirus in Early Life

**DOI:** 10.3389/fimmu.2014.00552

**Published:** 2014-10-30

**Authors:** Ariane Huygens, Nicolas Dauby, David Vermijlen, Arnaud Marchant

**Affiliations:** ^1^Institute for Medical Immunology, Université Libre de Bruxelles, Charleroi, Belgium; ^2^Faculty of Pharmacy, Université Libre de Bruxelles, Brussels, Belgium

**Keywords:** cytomegalovirus, fetus, infant, γδ T lymphocytes, αβ T lymphocytes, B lymphocytes, NK cells, dendritic cells

## Abstract

Cytomegalovirus (CMV) is the most common congenital infection and is the leading non-genetic cause of neurological defects. CMV infection in early life is also associated with intense and prolonged viral excretion, indicating limited control of viral replication. This review summarizes our current understanding of the innate and adaptive immune responses to CMV infection during fetal life and infancy. It illustrates the fact that studies of congenital CMV infection have provided a proof of principle that the human fetus can develop anti-viral innate and adaptive immune responses, indicating that such responses should be inducible by vaccination in early life. The review also emphasizes the fact that our understanding of the mechanisms involved in symptomatic congenital CMV infection remains limited.

## Introduction

Cytomegaloviruses (CMV) belong to the betaherpesvirus family and establish a lifelong infection in their host. A number of species can be infected by CMV but each virus has adapted to its host. Specifically, the virus infecting humans is unable to establish a productive infection in animals (1). This review will be focused on human CMV infection and data from mouse or non-human primate CMV infection will be referred to when necessary. During the chronic phase of the human CMV infection, the virus becomes latent and reactivates sporadically. Viral excretion during the chronic phase can be related to the reactivation of the latent virus or to superinfections with new CMV strains (2).

Human CMV is the most common congenital infection, affecting 0.2–2.2% of all live births. Congenital CMV infection is the leading non-genetic cause of neurological defects, including mental retardation, cerebral palsy, and hearing impairment (3, 4). Approximately 10% of CMV-infected newborns present clinical signs and symptoms including small birth weight, petechiae, jaundice, hepatosplenomegaly, microcephaly, and seizures. Symptomatic infections are more common and more severe in newborns infected during the first trimester of gestation. The prognosis of symptomatic newborns is poor, as more than 90% have long-term sequelae and mortality rates among the most affected ranges from 10 to 30%. Among asymptomatic newborns, 10–15% develops long-term sequelae during the first years of life, primarily hearing loss (5). Although 75–80% of infected newborns remain asymptomatic, they all excrete the virus for at least 5 years after birth (6), indicating a limited control of viral replication in some organs.

Post-natal CMV infection is usually asymptomatic but severe disease, including respiratory distress syndrome and sepsis, can develop in very premature babies (7). However, long-term sequelae have not been reported following post-natal CMV infection. Prolonged viral excretion lasting for at least 2 years is also observed following CMV infection in young children whereas it only lasts several months in immunocompetent adults (8, 9). Together, these observations indicate that the control of CMV replication is limited during early life as compared to adult life and that this reduced viral control is associated with an increased risk of symptomatic infection in the fetus and in the very premature newborn. Studies of the immune response to CMV infection in early life have provided important information on the development of the human immune system and on the capacity of the fetus to mount anti-viral responses. However, our understanding of the pathogenesis of symptomatic infections and of the mechanism involved in the reduced control of viral replication remains limited. In this review, we summarize the available data on the innate and adaptive immune responses to CMV infection in early life as compared to adult life and we discuss their possible role in viral control and in the development of symptomatic infections.

## Innate Immune Responses: γδ T Lymphocytes and Natural Killer Cells

γδ T lymphocytes and natural killer (NK) cells are innate lymphocytes that develop and differentiate early during fetal life (reviewed by Vermijlen and Prinz in this Research Topic). γδ T cells express a receptor formed of a γ and a δ chain on their cell surface and are a prototype of unconventional T cells: they can react rapidly during the course of an immune response and they are major histocompatibility complex (MHC)-unrestricted. NK cells are innate lymphocytes that do not possess Rag recombinase-dependent rearranged antigen receptors and that are activated by the balance between activating and inhibitory invariant receptors.

### γδ T lymphocytes

About 15 years ago, the group of Julie Déchanet-Merville reported that γδ T cells participate in the response to CMV in solid organ transplanted patients and that γδ T cell expansions are associated with a better clinical outcome (10, 11). More recently, evidence of a protective role of γδ T cells was obtained in the mouse model of CMV infection (Myriam Capone and Julie Déchanet-Merville; Michael Mach and Thomas Winkler; 6th International γδ T cell conference, May 2014, Chicago, USA). The γδ T cells expanded by CMV in human adults are distinct from the phosphoantigen-reactive Vγ9Vδ2 T cells and are collectively called Vδ2 negative (Vδ2^−^) γδ T cells. Expansions of Vδ2^−^ cells are also observed in CMV-infected healthy adults and hematopoietic stem cell (HSC) transplanted patients (12–14). The expansion of T cells expressing particular combinations of Vγ and Vδ chains and the restriction of the CDR3 repertoires among these cells strongly suggest an antigen–ligand interaction. Confirming this prediction, a ligand of a CMV-reactive Vγ4Vδ5 T cell receptor (TCR) derived from an adult patient was recently shown to be the endothelial protein C receptor (EPCR) (15, 16). EPCR is a MHC-like molecule that binds lipids analogously to the antigen-presenting molecule CD1d. However, the Vγ4Vδ5 TCR binds EPCR in an antibody-like way, independently of lipids. The EPCR-dependent recognition of CMV-infected cells appears to be complex, as CMV infection does not induce EPCR expression itself but rather promotes the expression of a co-stimulatory complex promoting the detection of CMV infection by Vγ4Vδ5 cells (15). Importantly, although the Vγ4Vδ5 TCR binding EPCR was highly expanded in the single patient from whom it was derived, this particular clone has not been detected in other CMV-infected transplanted patients. This suggests that other γδ TCR, that may recognize other ligands, participate in the response to CMV infection in adults.

We have reported that congenital CMV infection elicits a marked response of fetal γδ T cells (17). The fetal γδ T cell expansions induced by CMV are restricted to Vγ9^−^ cells and express either Vδ1, Vδ2, or Vδ3 chains. So, in contrast to adults, CMV induces the expansion of Vδ2^+^ cells in the fetus. Of note, these fetal Vδ2^+^ cells are Vγ9^−^ and are therefore distinct from the “classical” phosphoantigen-responding Vγ9^+^Vδ2^+^ cells (17). As Vγ9^−^Vδ2^+^ T cells are very rare in CMV seronegative or CMV seropositive adults, it appears that CMV expands specific subsets of γδ T cells in the fetus (10, 18). Strikingly, the fetal γδ T cell population expanded by CMV is also enriched in a public TCR formed by the Vγ8 and the Vδ1 chains and containing the germ-line-encoded (no nucleotide addition between the V, D, and J gene segments) CDR3δ1-CALGELGDDKLIF/CDR3γ8-CATWDTTGWFKIF sequences. Cells expressing this public TCR were detected as early as 21 weeks of gestation (17). Recently, the same CDR3γ8-CATWDTTGWFKIF sequence was detected in healthy adults (of unknown CMV status) by high-throughput sequencing of the γ gamma chain repertoire (19). Until now, the public CDR3δ1 sequence detected in CMV-infected fetuses has not been detected in adults, either during primary of chronic CMV infection (Vermijlen et al., unpublished observation). These observations suggest important differences between the fetal and the adult γδ T cell repertoires (discussed in this Research Topic by Vermijlen and Prinz). This notion is supported by the fact that public CDR3δ2 and CDR3δ3 chains were also readily expanded in CMV-infected fetuses (17) (and Vermijlen et al., unpublished observation) but not in CMV-infected adults with solid organ transplantation (10, 12). Of note, the fetal public CDR3 sequences were completely germline-encoded, a characteristic that probably contributes to a higher probability of becoming a public CDR3δ chain (17).

The fetal γδ T cells induced by CMV infection express a type 1 effector phenotype, including the transcription factors T-bet and Eomes, the anti-viral cytokine IFN-γ, the cytolytic molecules perforin and granzymes as well as a range of NK receptors. Clones derived from CMV-infected newborns and expressing the public Vγ8Vδ1 TCR produce IFN-γ in a TCR/CD3-dependent manner upon incubation with CMV-infected target cells and show anti-viral activity (17). These data indicate that functional fetal γδ T cell responses can be generated during fetal life and suggest that this T cell subset could participate in the control of CMV replication. The role of γδ T cells may be particularly important in early life as this subset develops earlier than conventional αβ T cells during immune ontogeny in humans and mice. Accordingly, a central role of γδ T cells in immunity in early life has been demonstrated in a mouse model of intestinal parasite infection (20). However, as discussed later in this review, the possibility that γδ T cells as well as other components of the immune response, may contribute to fetal immunopathology cannot be excluded.

### Natural killer cells

Natural killer cells play an important role in the immune response to human CMV infection, as indicated by the multiple immune evasion strategies developed by the virus to escape NK cell control (21). As in other viral infections, the control of CMV replication by NK cells depends on the balance between activating and inhibitory receptors. In the mouse, NK cell depletion was shown to result in decreased viral control indicating that the balance is in favor of NK cell activation (22). In humans, CMV infection has been associated with the expansion of NKG2C^+^ NK cells in healthy subjects, in patients co-infected with HIV or hantavirus and in HSC-transplanted patients (23–28). NKG2C is an activating receptor recognizing HLA-E. Cell surface HLA-E expression is increased by the signal peptide of the human CMV UL40 protein, indicating that NKG2C^+^ NK cells may be stimulated by CMV-infected cells (29). More recently, a stable imprint of CMV infection has been described on the repertoire of killer cell immunoglobulin like receptors (KIRs) in healthy adults (30). Together, these observations indicate that specific subsets of NK cells are selectively expanded by human CMV infection. These cells may have a “memory” phenotype as it was described for mouse NK cells expressing the Ly49H receptor and recognizing the m157 mouse CMV protein (31, 32).

Expansions of NKG2C^+^ NK cells have also been observed in young children infected *in utero* with CMV and the frequencies of NKG2C^+^ NK cells were higher in symptomatic as compared to asymptomatic children (33). The potential role of NKG2C^+^ NK cells in congenital CMV infection was evaluated by assessing the number of copies of the *NKG2C* gene. About 4% of Japanese and European adults have a complete deletion of the *NKG2C* gene and 32–34% of the populations are heterozygous (34, 35). The *NKG2C* deletion frequency was comparable in children with congenital CMV infection and in uninfected controls (33). However, homozygous children had higher numbers of circulating NKG2C^+^ NK cells and total NK cells as compared to heterozygous children. These results suggest that NKG2C has an impact on NK cell homeostasis during CMV infection in early life but is not an essential determinant of CMV infection in exposed fetuses. This hypothesis is further supported by a recent report showing an association between deletions at the *NKG2C* locus and the frequency of differentiated CD57^+^ NK cells in CMV-infected children (36). The role of NK cells in the control of CMV infection in early life is supported by the report of a 3-month-old T^−^B^+^NK^+^ SCID infant who presented with a CMV gastroenteritis that resolved spontaneously without anti-viral treatment (37). NKG2C^+^ NK cells expressing a biased KIR repertoire were highly expanded in this patient and normalized when CMV viral load declined. Finally, CMV infection was shown to induce the maturation of NK cells after cord blood transplantation (28). Interestingly, this maturation is also observed in patients transplanted with NKG2C−/− cord blood cells, indicating that activating KIRs can also be involved (38).

## Innate Immune Responses: Dendritic Cells

Human dendritic cells (DCs) can be classified into myeloïd DCs (mDCs), whose main function is to prime and functionally polarize naive T lymphocytes, and plasmacytoïd DCs (pDCs) that produce high levels of type I interferons (IFNs). The interactions between CMV and human DCs have been studied *in vitro* using monocyte-derived DCs (moDCs) as a model of mDCs, CD34^+^ bone marrow progenitor cell-derived Langerhans cell-type DCs (LDCs) as well as differentiated mDCs and pDCs purified from peripheral blood. In the presence of CMV, adult mDCs, moDCs, and pDCs produce high levels of pro-inflammatory cytokines and type I IFNs (39–41). This activation of cytokine production by moDCs and LDCs is associated with a decreased expression of MHC and co-stimulatory molecules and with a reduced capacity to stimulate T lymphocytes, suggesting an immune evasion mechanism developed by the virus (42, 43). However, experiments involving blood mDCs purified from healthy adults indicated no interference with T cell stimulation (40). Inhibition of T cell activating properties was also observed in *in vitro* models of DC infection by mouse CMV. In contrast, CMV markedly activates mouse DCs *in vivo* and enables them to stimulate potent T cell responses to the virus [reviewed in Ref. (44)]. These discrepancies between *in vitro* and *in vivo* data have been attributed to differences in the nature of the DCs involved and to differences in the frequency of cells infected by the virus (44).

Dendritic cells are detected early during the ontogeny of the human immune system but their functional program is different from that of adult DCs (reviewed in this Research Topic by De Kleer et al.). Most of our current understanding of the biology of DCs in early life comes from studies of the interactions between pathogen-associated molecular patterns (PAMPs) with specific innate receptors. In contrast, how DCs interact with complex pathogens remains poorly understood. *In vitro* studies have shown that cord blood-derived moDCs and adult moDCs are equally susceptible to CMV infection (39). As observed following activation with PAMPs, cord blood moDCs infected with CMV produce low levels of IL-12, IFN-β, and IFN-λ1 as compared to adult cells (39). Similarly, cord blood pDCs produce lower amounts of IFN-α than adult pDCs upon *in vitro* incubation with CMV (45). In contrast, cord blood and adult moDCs infected with CMV produce similar levels of IFN-α and IFN-inducible genes, suggesting that mDCs may represent an important source of type I IFNs during CMV infection (39). The reduced capacity of newborn moDCs to produce IL-12, IFN-β, and IFN-λ1 in response to CMV infection probably involves a defective activation and nuclear translocation of IRF3. In contrast, the adult-like production of IFN-α may be related to IRF3-independent pathways (46). As discussed below, the reduced capacity of newborn DCs to produce IL-12 upon CMV infection may limit their capacity to promote the differentiation of Th1 cells and effector CD8 T cells. However, Th1 and effector CD8 T cell responses were shown to be induced independently of IL-12 in several experimental settings (47–50). Also, as indicated above, mouse studies have shown that the results obtained with *in vitro* models of CMV–DC interactions may not predict the responses of DCs to CMV infection *in vivo*.

## Adaptive Immune Responses: αβ T Lymphocytes

More than 20 years ago, studies of mouse CMV infection demonstrated the central role played by αβ CD4 and CD8 T lymphocytes in the control of CMV infection (51–53). Clinical studies of adult transplanted patients indicated that the development of potent T cell responses to CMV is associated with a better clinical outcome. Furthermore, adoptive transfer of CMV-specific T cell clones demonstrated that T lymphocytes efficiently control CMV replication in humans (54–59). CD8 T lymphocytes are the dominant T cell population in the brain of neonatally infected mice and play a central role in the control of CMV replication in this organ (60).

The characteristics of CMV-specific T cells have been abundantly studied during the chronic phase of the infection and these studies have significantly contributed to our understanding of anti-viral immunity in humans. A central feature of the T cell response to CMV infection is that it involves large clonal expansions occupying an important fraction of the T cell pool. A comprehensive antigen repertoire analysis indicated that CMV-specific T cells comprise on average 10% of the total CD4 and CD8 memory T cell compartments in chronically infected healthy adults (61). A second feature of the T cell response to CMV is that it includes a large proportion of cells expressing a late differentiation phenotype characterized by the loss of expression of the CD27 and CD28 co-stimulatory molecules, a phenotype that is not or rarely induced by other viral infections (62, 63). CMV-specific CD4 and CD8 T cells produce high levels of anti-viral cytokines, including IFN-γ, TNF-α, and MIP-1β, following *in vitro* stimulation with viral antigens and both subsets express high levels of perforin and granzyme and are cytolytic (64, 65).

As primary CMV infection is usually asymptomatic in immunocompetent adults, studies of the early phase of the infection are relatively scarce and primarily involve populations of transplanted patients and of pregnant women screened for CMV infection. Primary CMV infection in adults is characterized by a period of intense viral excretion in several body fluids that lasts on average 6 months (9, 66). Viremia is controlled more rapidly than viral excretion in body fluids but can persist several weeks to months despite the presence of CMV-specific T cells (67). Indeed, high frequencies of CMV-specific CD4 and CD8 T cells appear early following primary CMV infection and the peak of their response is followed by a drop in peripheral blood viral load (68–70). The intense viral replication and excretion taking place during the first months of primary CMV infection is associated with a reduced capacity of CMV-specific CD4 T cells to proliferate and to produce IL-2 (69, 71). The acquisition of CD4 T cell proliferative responses correlates with the control of viremia and with a reduced risk of mother-to-fetus transmission of CMV (67). These defective IL-2 and proliferative responses are associated with a lower functional avidity of CMV-specific CD4 T cells and with a lower production of the anti-viral cytokines IFN-γ and TNF-α as compared to the chronic stage of the infection (71). This state of functional unresponsiveness is associated with the upregulation of the inhibitory receptor program cell death (PD)-1 and inhibition of PD-1 increases CD4 T cell proliferative responses (71, 72). Together, these results indicate that the prolonged viral replication associated with primary CMV infection in adults is associated with a state of functional exhaustion of CD4 T cells similar to the one observed in patients with chronic HIV or hepatitis infections (73).

Historical studies detected weak or no T cell responses to congenital CMV infection. This defect lasted up to 5 years and was more severe in symptomatic children (6, 74). More recent studies using more sensitive assays demonstrated large responses of CD8 T cells following congenital or early post-natal CMV infection (8, 75–81). Fetal T cells activated *in utero* by CMV infection acquire a late differentiation phenotype (CD27^−^/CD28^−^) equivalent to the one acquired by adult T cells (75, 78, 79, 82). The differentiated CD8 T cell population contains large expansions of a restricted number of clones, strongly suggesting an antigen-specific rather than a bystander response (75). Direct evidence for antigen-specific responses was obtained using MHC class I tetramer staining and short-term peptide stimulation assays. CMV-specific fetal CD8 T cells produce anti-viral cytokines, including IFN-γ, TNF-α, and MIP-1β and express perforin-dependent cytolytic activity (75–77, 80). CD8 T cell responses to CMV have been detected in samples collected by cordocentesis as early as 22 and 28 weeks of gestation (75, 81). As single positive CD8 T cells can be detected in the human fetus by 14 weeks of gestation, it is likely that this subset participates in the defense against CMV infection from the second trimester of gestation (83). However, whether the magnitude and the quality of this response are equivalent to that of adults has not been studied in detail. Longitudinal studies of children infected *in utero* or soon after birth indicate that the frequency of CMV-specific IFN-γ-producing CD8 T cells as well as the repertoire of peptides they respond to increase during the first year of life (77, 78). As CMV-specific CD8 T cells were identified in these studies on the basis of their production of cytokines, it is unclear whether their increasing frequency with time after infection is related to cell multiplication or to an increased capacity to produce cytokines.

In contrast to CD8 T cell responses, very low or no CD4 T cell responses to CMV are usually detected in young children infected *in utero* or soon after birth (8, 74, 80). Similar results are obtained using either proliferation or cytokine production assays following *in vitro* stimulation with CMV antigens. These observations contrast with the rapid expansion of CMV-specific CD4 T cells following primary infection in adults (68–70, 80). It is unclear from the current literature whether the low responses observed in early life involve a defective expansion of CMV-specific CD4 T cells or an impaired differentiation of effector cells. Our recent studies indicate that congenital CMV infection is associated with large oligoclonal expansions of CD4 T cells expressing a Th1 phenotype and having a restricted capacity to produce effector cytokines as compared to adult cells (Huygens et al., unpublished observations). These results suggest that primary CMV infection in adults and in the fetus is associated with the functional exhaustion of virus-specific CD4 T cells and that the magnitude of this phenomenon is particularly intense during fetal life. A more intense functional exhaustion of fetal T cells could be related to their prolonged exposure to higher antigen loads, as observed in chronic viral infections in humans and animals (73, 84–89). Indeed, clinical studies indicate that higher CMV viral loads are detected in the blood and urine of congenitally infected newborns and post-natally infected infants as compared to adults (5, 67, 90–92). Further supporting this possibility, CMV-specific CD4 T cells responses increase during the first 2 years of life and the cessation of viruria was shown to be associated with the acquisition of proliferative T cell responses to CMV in congenitally infected children (6, 80).

It could be expected that the functional regulation of CD4 T cell responses to CMV in early life associated with high viral loads may also affect CD8 T cells. Indeed, our recent studies using MHC class I tetramer stainings indicate that CMV-specific fetal CD8 T cells have a reduced capacity to produce anti-viral cytokines as compared to adult cells (Huygens et al., unpublished observations). These results suggest that the increased frequency of cytokine-producing CD8 T cells observed during the first year after congenital CMV infection is related to an increase in their functional capacity (77, 78). Interestingly, a limited capacity of CD8 T cells to produce effector cytokines was also observed in children infected with HIV (93). Together, these results suggest that functional exhaustion may be an important mechanism limiting anti-viral αβ T cell responses in early life.

## Adaptive Immune Responses: B Lymphocytes

Studies of mouse CMV indicate that neutralizing antibodies protect against primary infection and reactivation by limiting viral dissemination in tissues and that transfer of B lymphocytes from immune animals protects immunocompromised hosts against lethal infection (94, 95). Antibodies also reduce CMV replication in the brain of neonatally infected mice and attenuate the morphological alterations caused by the virus (96). In humans, immunization with an adjuvanted CMV glycoprotein B vaccine primarily stimulating the production neutralizing antibodies protected against infection and viral replication (97, 98). On the other hand, CMV hyperimmune IgG (HIG) have been shown to provide protection against CMV-associated mortality in solid organ transplanted patients (99). HIG was also proposed for the prevention of mother-to-fetus transmission of CMV following primary infection during pregnancy but a recent randomized trial did not support this approach (100, 101). The impact of maternal antibodies on the post-natal transmission of CMV infection remains unclear. The avidity of breast milk IgG was shown to be inversely correlated with CMV milk viral load (102). However, intense transmission through breastfeeding is observed in areas where maternal CMV infection is universal and where babies are born with high levels of maternal antibodies (103). These observations suggest that maternal antibodies may not be sufficient to control the large viral load to which infants are exposed through breastfeeding.

Despite the evidence that antibodies are an important component of immunity against CMV, very little is known about the B lymphocyte response to CMV infection in humans. In chronically infected adults, an important part of the antibody response is directed against tegument proteins. On the other hand, the envelope glycoprotein B and UL128–131 pentamer complex are important targets of antibodies neutralizing CMV infection of fibroblasts and epithelial cells, respectively (104). Intriguingly, primary CMV infection is associated with the rapid acquisition of antibodies directed against tegument proteins whereas envelope glycoprotein-specific antibodies are only acquired after several months (105). These observations suggest that CMV has developed evasion mechanisms delaying the acquisition of neutralizing antibodies and that this may favor virus dissemination. Supporting this hypothesis, the rapid acquisition of neutralizing antibodies, particularly against epithelial cell infection, was shown to be associated with a decreased risk of mother-to-fetus transmission of CMV (106). The mechanism involved in the delayed production of CMV envelope glycoprotein-specific antibodies has not been elucidated. We recently reported that primary CMV infection in pregnancy has an important impact on memory B cell subsets. Indeed, large and sustained expansions of virus-specific activated (CD27^+^CD20^+^CD21^low^) and atypical (CD27^−^CD20^+^CD21^low^) memory B cells are detected in pregnant women diagnosed with primary CMV infection, particularly in those who were viremic at the time of analysis (107). Increased frequencies of activated and atypical memory B cells have also been detected during HIV infection and have been suggested to play an important role in the defective antibody responses observed in these patients (108). As observed in HIV-infected patients, atypical memory B cells have a phenotype of exhausted cells during primary CMV infection. This phenotype is characterized by the upregulation of multiple inhibitory receptors and a decreased production of cytokines (107). Further studies are needed to define the role of memory B cell subsets in the delayed production of envelope glycoprotein-specific B cells following primary CMV infection.

Very little is known about the B cell response to CMV infection in early life. Studies have shown that CMV-specific IgM are commonly detected in congenitally infected newborns (109–111). As maternal IgG are transferred through the placenta from the second trimester of gestation, the capacity of the fetus to develop a class-switched antibody response and to produce neutralizing antibodies cannot be assessed by serology. Direct analyses of fetal B cells will be required to address this important question. Several parameters are likely to limit B cell responses to CMV in early life. Firstly, vaccine studies indicated that the capacity of the newborn and the young infant to differentiate antibody-secreting plasma cells is lower than in adults. This limitation may be related to the intrinsic characteristics of B lymphocytes in early life and to a limited help provided by follicular helper T cells, as observed in animal models (112). Secondly, the intense viral replication observed in CMV-infected fetuses and young infants may induce an important functional exhaustion of B lymphocytes, as observed in adults (107). Finally, maternal antibodies transferred *in utero* may inhibit B cell responses to CMV in the fetus and in the young infant as they inhibit B cell responses to vaccines (reviewed by Niewiesk in this Research Topic). Study of the B cell response to CMV in early life should help us understand the potential contribution of this subset in viral control and may also provide important information on the ontogeny of B cell responses during fetal life.

## Concluding Remarks and Perspectives

The study of the immune response to CMV infection during fetal life and infancy demonstrated that cellular immune responses can be induced very early during the ontogeny of the human immune system (Table 1). Effector γδ T cells with anti-viral properties expand and differentiate during fetal life. Intriguingly, CMV expands different γδ T cell populations in the fetus and in the adult. This may be related to differences in the repertoire of γδ T cells in early life and/or in the ligands induced by CMV in fetuses and adults (discussed by Vermijlen and Prinz in this Research Topic). Studies should be conducted to better understand the physiological development of this important T cell subset in humans. CMV infection in early life also activates NK cells with anti-viral potential. γδ T cells and NK cells are the first lymphocytes to develop during fetal life and could therefore play a very important role in the control of CMV infection during the first months of gestation. CMV infection during fetal life induced the expansion and the differentiation of effector CD4 and CD8 T cells. Effector CD8 T cell responses are also triggered by congenital HIV, *Trypanosoma cruzi*, and *Toxoplasma gondii* infections, indicating the capacity of the fetal immune system to respond to a range of different intracellular pathogens (113–118). These observations have important implications for the immunization of newborns against intracellular pathogens. Indeed, if effector T cell responses can be induced *in utero* following natural infections, similar responses should be inducible by neonatal vaccination.

**Table 1:** **Immune response to CMV infection in early life: characteristics and gaps in knowledge**.

	Available information for early life infection	Gaps in knowledge
**INNATE IMMUNE RESPONSE**		
γδ T cells	- Expansion of fetal Vγ9^−^cells with public TCRs (17)	- Evolution of repertoire from fetal to adult life
	- Anti-viral activity *in vitro* (17)	- Identification of fetal γδ TCR ligands
		- Role in viral control and in immunopathology following congenital infection
NK cells	- Expansion of NKG2C^+^ cells in infants (23–28)	- Anti-viral activity of NK cells activated in early life
		- Role in viral control and in immunopathology following congenital infection
Dendritic cells	- pDCs: limited production of IFN-α (45)	- *In vivo* responses to early life infection
	- moDCs: limited production of IL-12, IFN-β, and IFN-λ1 and adult-type production of IFN-α and IFN-inducible genes (39)	- Role of DC subsets in promoting adaptive immune responses in early life
**ADAPTIVE IMMUNE RESPONSE**		
CD8 T cells	- Large clonal expansions of differentiated cells with anti-viral activity (8, 75–82)	- Functional capacity and repertoire diversity of fetal and infant cells
		- Persistence of memory cells following infection in early life
		- Role in viral control and in immunopathology following congenital infection
CD4 T cells	- Limited frequencies of cytokine-producing cells (8, 74, 80)	- Magnitude of response, repertoire diversity, and functional programing of cells
		- Mechanism underlying limited functional responses in the fetus and young infant and the emergence of responses in older children
		- Persistence of memory cells following infection in early life
		- Role in viral control and in immunopathology following congenital infection
B cells	- IgM responses (109–111)	- Development of effector cells and persistence of memory cells following infection in early life
		- Impact of maternal antibodies on fetal and infant B cell responses
		- Role in viral control following infection in early life
Regulatory T cells and erythroblasts	- No information available	- *In vivo* responses to early life infection
		- Role in controlling adaptive and innate responses in early life

The development of anti-viral effector T cells during fetal life also indicates that the fetal immune system is not systematically programed against inflammatory responses. Indeed, if the relative defect of cord blood DCs to produce type 1 IFNs and IL-12 can be extrapolated to DCs in the fetal organs, it does not appear to prevent the differentiation of pathogen-specific cytolytic CD8 T cells or Th1 cells (46). The differentiation of effector γδ T cells may not require interactions with DCs as they may be directly stimulated by the ligands expressed by infected cells in tissues (119). Identification of the ligand(s) of the public γδ TCR(s) expanded upon congenital CMV infection, will not only provide insight into the immune response toward CMV, but also into the general biology of γδ T cells. Once activated, γδ T cells, as well as NK cells, may in turn induce fetal DC maturation and thereby promote the differentiation of αβ effector T cells (120–122). Also, the development of effector T cell responses against intracellular pathogens such as CMV does not appear to be prevented by the regulatory T cells and erythroid cells that are detected at high frequencies during fetal life and at birth (123, 124). It will be important to determine whether these fetal regulatory cells are activated by CMV infection and whether they impact effector T cell responses.

Large oligoclonal expansions of fetal CD8 and CD4 T cells are detected following congenital CMV infection. It is unclear whether the repertoire diversity of the fetal T cells activated by CMV is similar to the one triggered by the virus in adults. In neonatal mice, the activity of terminal deoxynucleotide transferase (TdT), the enzyme responsible for template-independent nucleotide additions, is detected around 1 week of life and this activity correlates with the acquisition of an increased diversity of CDR3 sequences (125). In humans, TdT activity is detectable in the fetal thymus from the 20th week of gestation (126). As single positive thymocytes are already detected at 14th week of gestation, the diversity of the T cell repertoire may increase during the second trimester of gestation in humans as it does during the first week after birth in the mouse and this may influence the recognition of pathogen-derived peptides (125). Another dimension of the αβ T cell response to CMV in early life that has not been studied is the persistence of effector T lymphocytes. Longitudinal analyses of T cell repertoire in kidney transplanted adults showed that CMV-specific CD8 T cell clones that emerge during the early phase of primary infection are maintained at high frequencies during the next 5 years (127). Recent mouse studies indicated that the rapid proliferation and differentiation of effector CD8 T cells impair the development of memory cells in early life (128). It will be important to determine whether a similar process limits the maintenance of anti-viral T cells in early life in humans.

The development of effector and memory B cell responses in early life remains poorly understood. This gap of knowledge remains a major limitation to the improvement of neonatal immunization strategies (112, 129). The study of the B cell response to congenital CMV infection could provide important insight into the molecular mechanisms controlling the differentiation and the survival of B lymphocytes in early life.

As discussed above, our recent observations indicate that effector CD8 and CD4 T cells are functionally exhausted during primary CMV infection and suggest that this phenomenon is particularly intense in early life. This intense degree of functional exhaustion may be related to a more intense CMV replication in the fetus and young infant than in the adult. A similar association between functional exhaustion and intense viral excretion was observed following primary CMV infection of juvenile rhesus macaques (130). As described in animal models of chronic viral infections, the reduced anti-viral properties of exhausted T lymphocytes may, in turn, limit their capacity to control CMV replication. Directly assessing the impact of T cell exhaustion on CMV replication will require an animal model such as the one recently described in rhesus macaques (130). Of note, mouse studies have shown that CMV replication in the salivary glands and viral excretion in saliva are primarily controlled by CD4 T lymphocytes (52). Defining whether human CD4 T cells are also central to the control of viral replication in salivary glands and in the kidney has potential implications for the design of vaccines aiming at reducing CMV transmission.

Finally, our understanding of the pathogenesis of symptomatic congenital CMV infection in humans remains very limited. However, mouse studies have provided important insights in the potential mechanisms involved and suggest that the immune response to CMV may play a dual role in the neurological pathology of neonatal mice by controlling CMV replication but also by inducing inflammatory responses promoting developmental abnormalities [reviewed in Ref. (131)]. The few histopathological studies of symptomatic human fetuses indicate that severe brain lesions, including microglial nodules, necrosis, and polymicrogyria, are associated with high tissue CMV viral load and with intense infiltration of CD8 T lymphocytes. Tissue necrosis may involve CD8 T cell-dependent immunopathology but may also be caused by the hypoxia resulting from the dysfunction of the CMV-infected placenta (132). Biomarker studies indicate that severe brain damage in the fetus is correlated with peripheral blood viral load and with the intensity of the immune response, measured as the plasma levels of β2-microglobulin and anti-CMV IgM (91). Further studies are needed to increase our understanding of the role of the immune system in the pathogenesis of symptomatic congenital CMV infection and to identify markers predicting the development of neurological complications *in utero* and after birth. Ganciclovir is recommended for the prevention of long-term neurological sequelae in symptomatic infants (133). If the inflammatory response induced by CMV plays a role in the development of these sequelae, anti-inflammatory agents may complement this therapeutic strategy (131). However, if research efforts are needed to better understand the pathogenesis of congenital CMV infection and to develop better therapies for affected children, the control of the disabilities caused by congenital infection will require the development of an efficient vaccine preventing the transmission of the virus to the fetus.

## Conflict of Interest Statement

The authors declare that the research was conducted in the absence of any commercial or financial relationships that could be construed as a potential conflict of interest.
